# Neuromesodermal progenitors and the making of the spinal cord

**DOI:** 10.1242/dev.119768

**Published:** 2015-09-01

**Authors:** Domingos Henrique, Elsa Abranches, Laure Verrier, Kate G. Storey

**Affiliations:** 1 Instituto de Medicina Molecular and Instituto de Histologia e Biologia do Desenvolvimento, Faculdade de Medicina da Universidade de Lisboa, Avenida Prof. Egas Moniz, Lisboa 1649-028, Portugal; 2 Division of Cell & Developmental Biology, College of Life Sciences, University of Dundee, Dow Street, Dundee DD1 5EH, UK

**Keywords:** Neuromesodermal progenitors, Wnt, FGF, Bipotent cells, Neural induction, Spinal cord, Stem cells

## Abstract

Neuromesodermal progenitors (NMps) contribute to both the elongating spinal cord and the adjacent paraxial mesoderm. It has been assumed that these cells arise as a result of patterning of the anterior neural plate. However, as the molecular mechanisms that specify NMps *in vivo* are uncovered, and as protocols for generating these bipotent cells from mouse and human pluripotent stem cells *in vitro* are established, the emerging data suggest that this view needs to be revised. Here, we review the characteristics, regulation, *in vitro* derivation and *in vivo* induction of NMps. We propose that these cells arise within primitive streak-associated epiblast via a mechanism that is separable from that which establishes neural fate in the anterior epiblast. We thus argue for the existence of two distinct routes for making central nervous system progenitors.

## Introduction

**Fig. 1:**
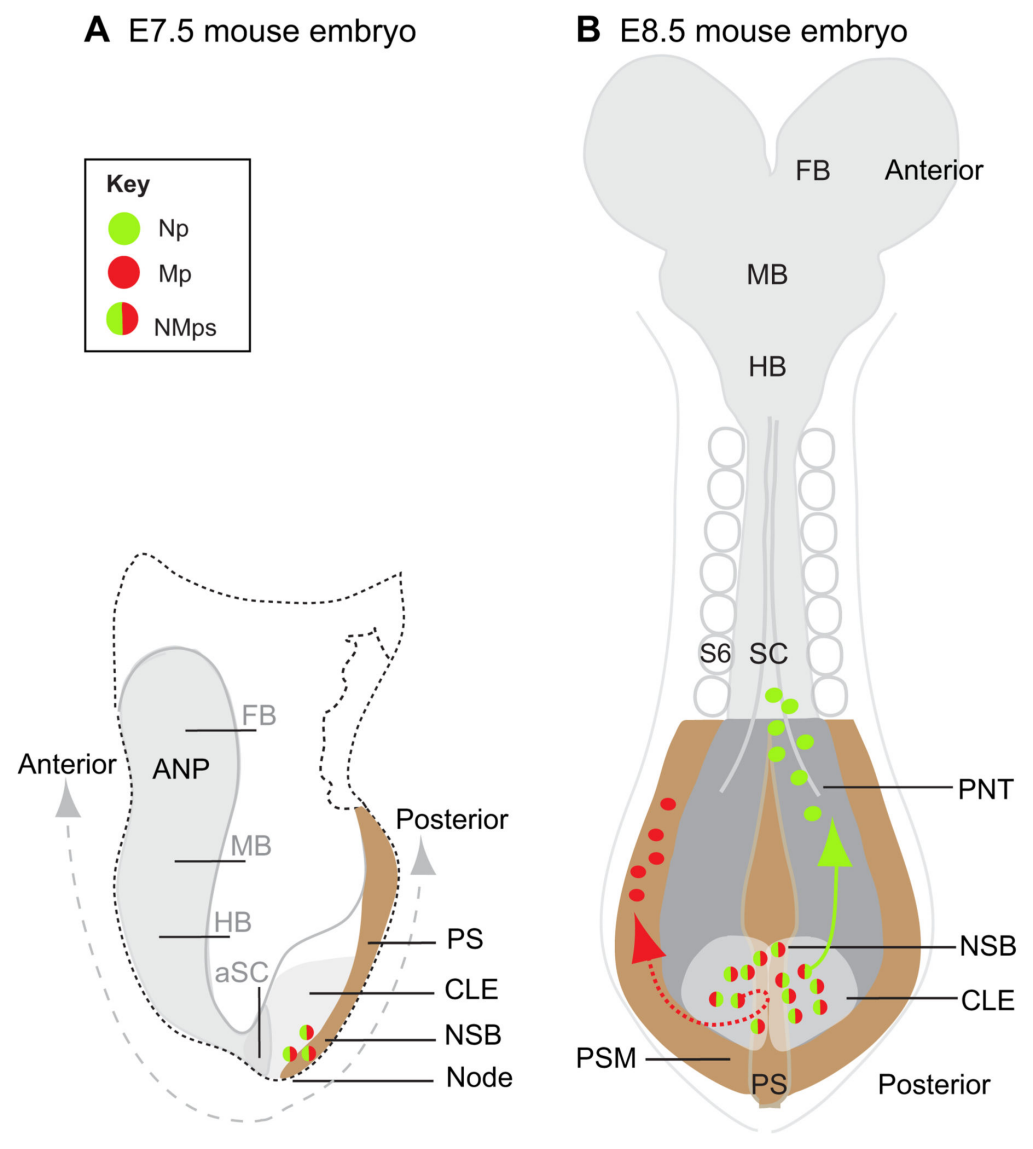
Key features of the developing CNS and neuromesodermal progenitors in the embryo. Schematics of E7.5 (A) and E8.5 (B) mouse embryos indicating cell populations that give rise to the CNS. At E7.5, the anterior neural plate (ANP) consists of prospective forebrain (FB), midbrain (MB), hindbrain (HB) and some anterior spinal cord (aSC) progenitors; more posterior spinal cord arises from neuromesodermal progenitors (NMps; red/green), which are located in the node-streak border (NSB) in the anterior primitive streak (PS; brown) and in the adjacent caudal lateral epiblast (CLE; light grey). At E8.5, NMps have given rise to new neural progenitors (Np; green), which contribute to the CLE (light grey) and then the preneural tube (PNT; dark grey), and to new mesoderm progenitors (Mp; red), which contribute to presomitic mesoderm (PSM; brown). The rostralmost position reported for Nps generated by NMps is the ventral region of the anterior spinal cord approximately at the level of somite 6 (S6).

The vertebrate central nervous system (CNS) is first manifest as an ovoid region of thickened epiblast cells in front of the organiser/anterior primitive streak. This region is known as the anterior neural plate (Fig. 1). Fate-mapping studies in a range of vertebrate species all show that the forebrain forms in the rostralmost part of this region, whereas more posterior regions of the CNS (midbrain and hindbrain) arise from cells positioned closer to the primitive streak. The position of the prospective hindbrain/spinal cord is more variable between species; in the chick, for example, this is located closest to the primitive streak (Spratt, 1952), whereas in the mouse embryo some laterally positioned epiblast cells also move medially to contribute to posterior neural tissue (Lawson and Pedersen, 1992).

**Fig. 2:**
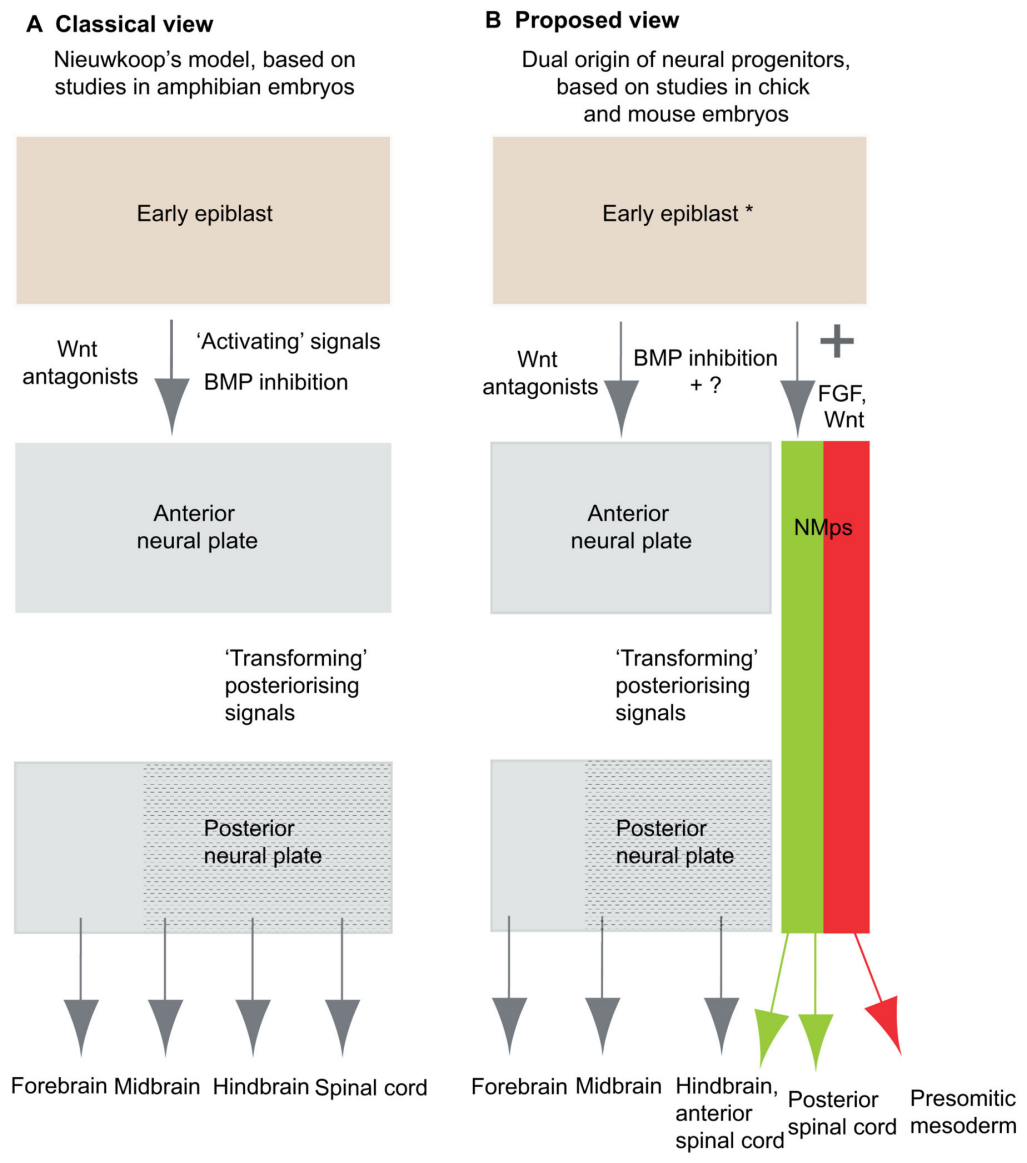
Comparison of neural induction models. (A) Prevailing view of vertebrate neural induction based on work in the amphibian embryo. This model, derived from Nieuwkoop's ‘activation-transformation’ hypothesis, involves the induction of an initial anterior neural plate that is subsequently regionalised by posteriorising signals to form posterior neural plate. (B) Proposed view of neural induction involving a dual origin of neural progenitors. In this model, epiblast cells (which in chick may have entered an unstable ‘preneural’ state, indicated by the asterisk) acquire neural fate either in the anterior neural plate (which is then progressively subdivided as proposed by Nieuwkoop) or via the induction of primitive streak-associated neuromesodermal progenitors (NMps), which contribute progenitors to anterior and posterior spinal cord and to flanking presomitic mesoderm (see text for details).

The prevailing view of vertebrate neural induction derives largely from work in the amphibian embryo. This proposes that initial induction of the anterior neural plate is followed by the formation of more posterior neural regions via patterning of this anterior tissue with posteriorising signals (to form posterior neural plate) (Fig. 2A). This view was first formulated in the so called ‘activation-transformation’ hypothesis proposed by Nieuwkoop (Nieuwkoop, 1952; Nieuwkoop and Nigtevecht, 1954), in which ‘activation’ involved the induction of anterior neural tissue and ‘transformation’ implied its patterning to more posterior character (Fig. 2A). This was subsequently substantiated at the molecular level with the discovery that inhibition of bone morphogenetic protein (BMP) signalling promoted the formation of anterior neural tissue (with forebrain character), which could then be patterned by posteriorising signals, such as retinoic acid (RA), Wnt and fibroblast growth factors (FGFs).

The molecular basis for this ‘activation’ step is not without controversy when extended to amniote embryos. Although inhibition of BMP signalling promotes neural fate in the mouse embryo, for example (Di-Gregorio et al., 2007), BMP inhibition alone is insufficient to induce neural tissue in the chick extraembryonic epiblast (Stern, 2006). This might reflect differences in experimental assays, especially the timing of manipulations, and/or the operation of species-specific mechanisms. It is also now recognised that neural induction is a complex multistep process. This includes roles for FGF signalling as the mediator of an early unstable‘preneural’ state in the chick embryo, which is then stabilised by further (yet to be identified) signals (Stern et al., 2006). However, it should be noted that some studies have not found a requirement for FGF/Erk signalling during neural differentiation, for example in embryonic stem cells (ESCs) and epiblast-derived stem cells (EpiSCs) (Greber et al., 2010, 2011; Ozair et al., 2013b; Hamilton and Brickman, 2014). Wnt signalling, or its antagonism, is also variably implicated in this ‘activation’ step in different species. Wnt, FGF and RA signalling then subsequently act as local posteriorising factors, while Wnt antagonism promotes anterior/forebrain identity. Detailed reviews of neural induction are provided elsewhere (Stern, 2005, 2006; Ozair et al., 2013a; Andoniadou and Martinez-Barbera, 2013). However, a common premise here is that the acquisition of neural fate starts with induction of the anterior neural plate, and that this is achieved as a result of events in the anterior epiblast, which gives rise to the entire CNS.

The discovery of a bipotent neuromesodermal progenitor (NMp) that contributes to both the spinal cord and paraxial mesoderm in the mouse embryo (Tzouanacou et al., 2009) has now raised the possibility that some posterior neural tissue is generated independently of the mechanism(s) that induces the anterior neural plate. The idea that the posterior spinal cord arises from progenitor cells with a neuromesodermal potential was proposed as long ago as 1884, based on morphological observations (Kölliker, 1884), and there has been a long-running debate about whether head, trunk and tail regions of vertebrate embryos are induced by distinct mechanisms (Handrigan, 2003; Stern et al., 2006). In more recent years, fate-mapping studies of groups of cells in mouse and chick embryos at late primitive streak to tailbud stages (Brown and Storey, 2000; Iimura and Pourquié, 2006; Cambray and Wilson, 2007; Olivera-Martinez et al., 2012) have localised this NMp cell population to the caudal lateral epiblast (CLE; also known as the stem zone or caudal neural plate in chick) and adjacent node-streak border (NSB) (Fig. 1). Recent studies have also demonstrated that mouse ESCs and EpiSCs, as well as human ESCs, can be directed to form NMps *in vitro* (Gouti et al., 2014; Tsakiridis et al., 2014; Turner et al., 2014a; Denham et al., 2015; Lippmann et al., 2015; Tsakiridis and Wilson, 2015), raising the possibility of exploring the potential therapeutic use of NMps (see Box 1). These cells can be passaged to some extent, and establishment of *in vitro* derivation protocols has facilitated their characterisation, allowing genomescale analyses and their ready manipulation. Indeed, NMps derived from a critical mass of ESC-derived epiblast-like cells can form a ‘gastruloid’ that produces both a neural and an emerging mesodermal cell population (Turner et al., 2014a, b; van den Brink et al., 2014), lending support to the idea that NMps persist during body axis elongation, providing new neural and mesodermal tissues over an extended period.

Clearly, the existence of NMps challenges traditional notions of the formation of three germ layers (ectoderm, mesoderm and endoderm) and subsequent neural cell fate assignment from within the ectoderm. In the prevailing view of neural induction, NMps are derived from the anterior neural plate, and the setting aside of these cells from within this neuroepithelium might then be considered a patterning event dependent on prior formation of anterior neural tissue (Fig. 2A). An alternative hypothesis proposed here (Fig. 2B) is that the induction of NMps close to and within the primitive streak involves a distinct step that is independent of the formation of anterior neural tissue.

Here, we review the evidence for NMps, focusing largely on data from amniote embryos, and consider their molecular characteristics and the signals that induce them *in vivo* and *in vitro*. We also evaluate experiments in the embryo, which suggest that anterior and posterior neural tissue can form independently. Finally, we review lineage data and gene regulatory interactions to speculate on the point at which anterior-posterior pattern and neural fate are established in the early epiblast and how this relates to the induction of NMps.

## Evidence for NMps

The most compelling evidence for dual-fated NMps comes from a retrospective clonal lineage analysis carried out in the elongating mouse embryo (Tzouanacou et al., 2009). This study exploited the random labelling of single cells that takes place when a mutant *laacZ* transgene reverts at low frequency to a functional *lacZ* gene, the expression of which marks the single revertant cell and all its progeny (constituting a clone) (Bonnerot and Nicolas, 1993). The analysis of labelled clones revealed the existence of cell lineages that contribute to both paraxial mesoderm and the spinal cord, and that also include cells located in the E10.5 chordoneural hinge, the only tailbud cell population with self-renewing properties (Cambray and Wilson, 2007; McGrew et al., 2008). This suggests that individual cells (NMps) are retained posteriorly (in the tailbud) and generate cells that can contribute to neural or mesodermal lineages as the body axis extends. However, some other clones containing neural and mesodermal cells lacked labelled cells in the chordoneural hinge. This indicates that NMps have a tendency to differentiate and, for this reason, these cells may be most accurately referred to as long-term NMps rather than neuromesodermal or axial stem cells (Tzouanacou et al., 2009). Indeed, the number of neural/ mesodermal clones found in embryos assessed at different stages of development (gastrulation, organogenesis and tailbud stages) varied, with more clones at the organogenesis stage (E8.5), when the trunk is being generated (Tzouanacou et al., 2009). One interpretation of these findings is that NMps are an evolving cell population that arises early in development and which increases and then decreases during the generation of the body axis.

Retrospective clonal analysis does not directly indicate the location of NMps in the embryo. However, fate-mapping studies in which small groups of cells were labelled have helped to identify regions where NMps may reside in the embryo. In the chick, dye labelling of groups of one to three cells in the CLE identified a region close to the primitive streak that is able to contribute to both neural and mesodermal lineages at early somite stages (Brown and Storey, 2000). Labelling cells in a similar position by electroporation of plasmids driving fluorescent protein expression in chick embryos confirmed this finding (Iimura and Pourquié, 2006). In the mouse embryo, grafting GFP-expressing cells of the NSB to the same position in wild-type embryos further confirmed this region of the primitive streak, as well as the CLE, as a site containing cells that are able to contribute to neural and mesodermal lineages (Cambray and Wilson, 2007). However, NSB-derived cells additionally contributed to notochord, and studies of both mouse and chick embryos in which single cells were dye labelled in the node have demonstrated that individual cells can contribute to multiple lineages, including to paraxial mesoderm and neural tissue or to paraxial mesoderm and notochord, as well as to notochord alone (Selleck and Stern, 1991; Forlani et al., 2003; Wilson et al., 2009). The mouse NSB therefore appears to be a more heterogeneous population than the CLE.

Further persuasive evidence for the existence of NMps comes from the ability to derive cells with these characteristics from pluripotent stem cells via the approach of *in vitro* differentiation (discussed in detail below). Recent work using this approach also provides evidence strongly suggesting that single cells with the molecular hallmarks of NMps can give rise to clones containing both neural and mesodermal progenitors (Tsakiridis and Wilson, 2015).

## Defining NMps

**Fig. 3:**
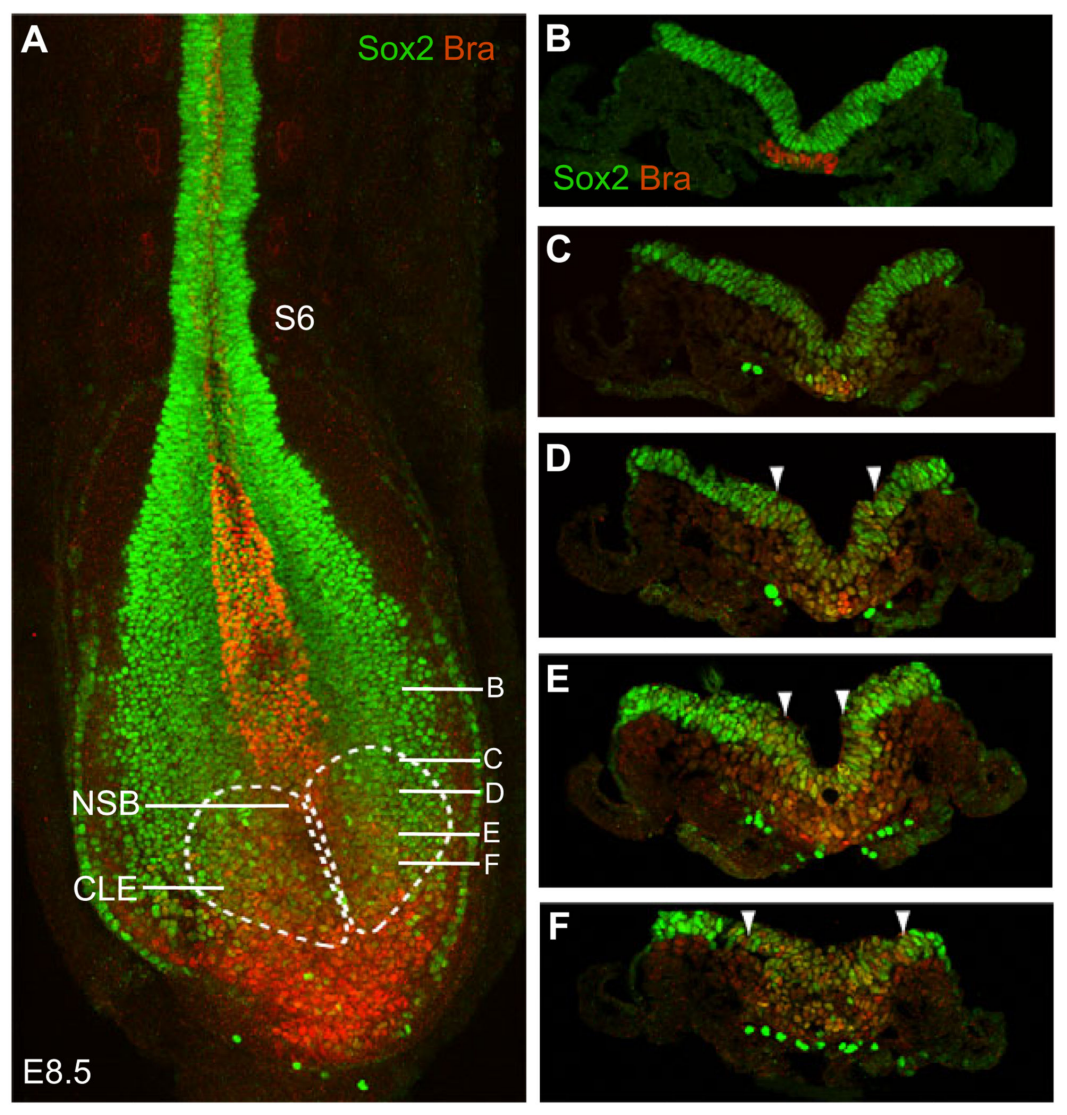
Sox2 and brachyury co-expressing cells in the CLE and primitive streak. (A) Confocal maximum intensity projection of the posterior end of an E8.5 (6-somite, S6) mouse embryo labelled with antibodies against Sox2 (green) and brachyury (Bra; red). Note the double-labelled cells in the CLE (white dashed lines) and NSB. (B-F) Transverse sections at the levels indicated in A. Note the double-labelled cells in the primitive streak and adjacent CLE (between the arrowheads). Sox2 is also detected in large, ventrally located migrating germ cells.

Unique molecular markers for NMps are currently lacking. In recent studies, however, co-expression of the early mesodermal marker brachyury (T/Bra) and the neural progenitor marker *Sox2* has been used to identify these cells in the epiblast associated with the primitive streak (Fig. 3). In mouse embryos, Bra/Sox2 co-expression in the CLE/NSB at E8.5 appears to correlate with the position of NMps, as determined by fate-mapping experiments in which defined cell groups from GFP-expressing embryos are transplanted to wild-type embryos (Tsakiridis et al., 2014). Furthermore, genetic fate-mapping of *Bra*-expressing cells (using *Bra*-Cre lines) has indicated that these cells contribute significantly to the spinal cord (see below), confirming that *Bra* is indeed expressed in cells with neural potential, in addition to its well-known expression in prospective mesoderm (Perantoni et al., 2005; Anderson et al., 2013; Imuta et al., 2013; Chalamalasetty et al., 2014; Garriock et al., 2015).

In the NMp-containing epiblast region, *Sox2* expression is driven by a unique enhancer element (termed N1), which, importantly, is distinct from that (N2) promoting *Sox2* expression in ESCs and subsequently in the anterior epiblast (Uchikawa et al., 2003; Iwafuchi-Doi et al., 2011, 2012). In the mouse embryo, a transition from N2 to N1 enhancer activity in cells close to the primitive streak appears to mark the epiblast cell population that will form the posterior nervous system/CLE (Takemoto et al., 2006; Iwafuchi-Doi et al., 2011, 2012). However, it should be noted that the N1 enhancer is first activated along the primitive streak and its activation domain then spreads laterally into the CLE (Yoshida et al., 2014). It is also apparent that, although all CLE cells express *Sox2*, only a subset co-express *Sox2* and *Bra* in this region, indicating that N1 enhancer activity is not unique to NMps. Other genes, including *Nkx1.2* (*Sax1*) (Spann et al., 1994; Schubert et al., 1995; Delfino-Machin et al., 2005) and the chick achaete-scute gene homologue *Cash4* (Henrique et al., 1997; Akai et al., 2005) are also expressed across the CLE and into the preneural tube (PNT) (Fig. 1) and thus may identify both NMps and recently generated neural progenitors. A population of cells co-expressing Bra/Sox2 has also been identified at late stages in the tailbud of chick and human embryos (Olivera-Martinez et al., 2012). Dye labelling of this late cell group in the chick demonstrated that it also contributes to the neural tube and paraxial mesoderm (Olivera-Martinez et al., 2012). This is consistent with the continued activity of NMps during mouse axis elongation deduced by Tzouanacou et al. (2009).

## Signals directing NMp generation

**Fig. 4:**
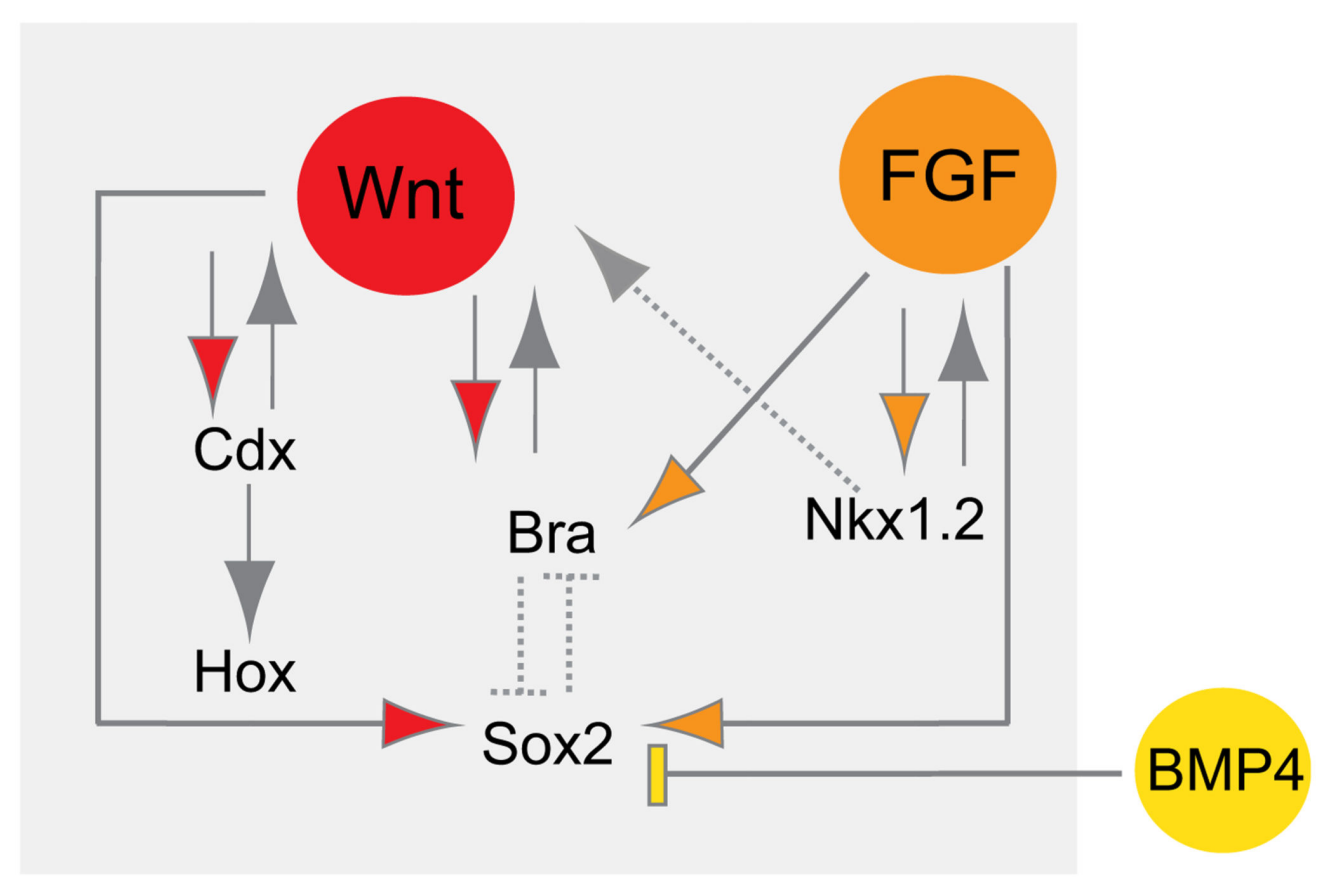
Key signals and transcriptional networks regulating NMps. FGF and Wnt signals provided by the primitive streak and CLE induce the expression of *Bra* and the *Sox2* (N1) enhancer, and Bra in turn promotes Wnt signalling. FGF signalling also promotes expression of Nkx1.2 (Sax1), and this transcription factor in turn induces *Fgf8* transcription; it also indirectly promotes Wnt signalling by inhibiting expression of the repressor *Tcf3* [indicated with a dotted line as evidence comes from P19 cells (Tamashiro et al., 2012)]. Wnt signalling induces the expression of Cdx genes, which act both to promote Wnt signalling and to regulate caudal Hox gene expression. *Sox2* transcription is also repressed by BMP signalling delivered by epiblast cells posterior and lateral to the CLE and so defines the domain within which NMps can arise. The co-expression of Sox2 and Bra is a central feature of NMps and there is some evidence that they are mutually repressive (indicated by dotted inhibition symbols). For example, *Sox2* mRNA expression is high in *Bra* mutant NMps in which Wnt is activated (Gouti et al., 2014); in the frog, T-box genes directly repress *Sox2* (Gentsch et al., 2013); and in the mouse the presomitic mesoderm gene *Tbx6* represses *Sox2* via the N1 enhancer (Li and Storey, 2011; Takemoto et al., 2011). Conversely, Sox2 N1 loss (in a *Sox3* null background) increases the ingression of cells to form presomitic mesoderm (Yoshida et al., 2014), suggesting that Sox2 normally restrains this Bra-induced activity; Sox2 also binds the *Bra* promoter in ESC-derived neural progenitors and Sox2 overexpression represses *Bra* in a Wnt-driven mesodermal differentiation assay (Zhao et al., 2004; Thomson et al., 2011). This mutual repression between Sox2 and Bra might underpin the creation of a state in which cells are poised to adopt either neural or mesodermal cell fate.

Taken together, the findings above strongly suggest that NMps in the embryo co-express *Sox2* and *Bra*. In recent years, a number of *in vivo* and *in vitro* studies have revealed how the expression of these transcription factors is regulated by the Wnt, FGF and BMP signalling pathways. These studies have also uncovered regulatory links between these pathways and further key transcription factors involved in the generation and patterning of the posterior body. Overall, a complex gene regulatory network involving cross-regulation of transcription factors and signalling pathway components appears to define the NMp cell state (Fig. 4).

### Insights from the embryo

Wnt and FGF signalling have long been known to promote posterior neural character in vertebrate embryos (e.g. Cox and Hemmati-Brivanlou, 1995; Lamb and Harland, 1995; Storey et al., 1998; Kiecker and Niehrs, 2001; Nordström et al., 2002) and it is therefore not surprising that these signals are associated with NMp formation. Inputs from both FGF and Wnt signalling are required to promote *Sox2* N1 enhancer activity in the CLE (Takemoto et al., 2006). Candidate molecules include Fgf4, Fgf8, Wnt3a and Wnt8a/c, which are provided locally by cells in the anterior primitive streak and adjacent epiblast.

Wnt3a is also known to promote *Bra* expression (Yamaguchi et al., 1999; Martin and Kimelman, 2008; Savory et al., 2009) and to orchestrate the genetic network controlling paraxial mesoderm formation (Nowotschin et al., 2012; Chalamalasetty et al., 2014). Loss of this ligand has dramatic effects on the assignment of mesodermal versus neural cell fates, both in mouse (Takada et al., 1994; Yoshikawa et al., 1997; van de Ven et al., 2011) and zebrafish (Martin and Kimelman, 2012) embryos, causing the formation of ectopic neural tissue and loss of posterior mesodermal structures. By contrast, excess Wnt activity due to the expression of an activated form of β-catenin in zebrafish embryos causes the opposite phenotype, promoting mesodermal over neural fate. This led to a model in which Wnt signalling regulates fate choices of bipotent NMps, repressing neural fates and promoting mesodermal development (Martin and Kimelman, 2012).

However, in *Tbx6* mouse mutants, in which prospective mesoderm cells ingress but form ectopic neural tubes, *Wnt3a* expression persists despite the failure to make mesoderm; this condition indicates that Wnt signalling does not inhibit neural fate. Instead, these results suggest that the primary role of Wnt3a is to maintain NMps, which then form neural tissue when mesoderm differentiation fails (Takemoto et al., 2011). This interpretation is supported by a recent analysis of transgenic mice in which constitutive Wnt signalling was achieved by overexpression of dominant stabilised β-catenin directed by a *Bra*-Cre driver (Garriock et al., 2015; and see Jurberg et al., 2014). In such embryos, cells with active Wnt/β-catenin differentiate primarily into mesoderm but can still contribute to the neural tube. However, in both studies, despite making some neural tissue, such embryos soon stop elongating and accumulate a mass of unsegmented mesoderm at the posterior end. These findings suggest that Wnt functions to maintain NMps and that prolonged exposure to Wnt can bias these cells towards the mesoderm fate. In another transgenic mouse line described by Jurberg et al. (2014), ectopic Wnt3a was driven by a *Cdx2* enhancer in the posterior epiblast, which acts before Bra expression. In these Cdx2P-Wnt3a embryos, no neural tube was formed and mesoderm differentiation was partially blocked. Furthermore, these high Wnt3a-expressing cells appeared to remain undifferentiated in an early epiblast-like state, suggesting that premature Wnt signalling interferes with the establishment of the NMp cell state.

Together, these experiments indicate that the timing and duration of Wnt activity are important parameters for the induction and maintenance of NMps and that although prolonged Wnt signalling can bias cells towards mesoderm fate, Wnt activity is not incompatible with acquisition of the neural progenitor state. Indeed, sustained β-catenin activity has a further role in NMp-derived neural and mesodermal progenitors, in which it now blocks the progression of differentiation (Garriock et al., 2015). This is consistent with the expression and activity of Wnt8a/c in neural progenitors leaving the CLE (Olivera-Martinez and Storey, 2007) and with previous reports that Wnt signalling promotes proliferation in the established neural tube (Megason and McMahon, 2002). These findings thus indicate that Wnt signalling has sequential roles in NMps and in their derivatives.

As noted above, FGF signalling is implicated in neural induction and posteriorisation, but it is also involved in mesodermal induction (reviewed by Stern, 2005) and in the direct regulation of *Bra*, as shown first in the frog embryo (Isaacs et al., 1994). FGF signalling also promotes the expression of many genes expressed in the CLE (*Nkx1.2*, *Cash4* and *Wnt8c*) and inhibits the progression of differentiation in this caudal region (reviewed by Wilson et al., 2009). The loss of both Fgf4 and Fgf8 specifically in late-gastrula mouse embryos has further demonstrated a direct requirement for FGF signalling for the production of posterior neural and mesodermal tissues (Naiche et al., 2011; Boulet and Capecchi, 2012). These studies found no increase in cell death or defects in cell proliferation or migration, suggesting that FGF signalling is important for maintenance of the NMp state.

It is also clear from many studies that FGF and Wnt signalling operate in a positive-feedback loop in posterior tissues. For example, Wnt3a is required for *Fgf8* expression in the primitive streak/tailbud (Aulehla et al., 2003; reviewed by Wilson et al., 2009). The transcription of *Sox2* (but not *Sox2* N1 enhancer activity) is also inhibited by BMP signalling, which restricts *Sox2* transcripts to the CLE/NSB (Takemoto et al., 2006) and so helps to define the domain within which NMps can arise (Fig. 4).

Finally, there are cross-regulatory links between these signalling pathways and key transcription factors at work in the CLE (Fig. 4). Nkx1.2, for instance, is known to promote *Fgf8* transcription in the chick embryonic body axis (Sasai et al., 2014), and also to repress *Tcf3* in P19 cells, thereby facilitating Wnt-mediated upregulation of *Bra* in these cells (Tamashiro et al., 2012). Reciprocal expression of *Tcf3* with that of *Nkx1.2* and *Bra* in the early mouse embryo suggests that this regulatory relationship holds *in vivo* (Merrill et al., 2004). Wnt signalling is required for the expression of Cdx genes (*Cdx1*, *2* and *4*), which are key mediators of caudal Hox gene expression (Fig. 4) (van den Akker et al., 2002; Nordström et al., 2006; Young et al., 2009; van de Ven et al., 2011; Mazzoni et al., 2013). Hox gene expression determines anterior to posterior identity, with genes located 3′ of the Hox gene cluster expressed in anterior regions, whereas more 5′ Hox genes confer progressively more posterior identity (Mallo and Alonso, 2013). Indeed, by regulating the expression of these transcription factors and of key components of the Wnt, FGF and RA signalling pathways, Cdx genes are thought to integrate the generation and patterning of the posterior body axis (Savory et al., 2009; Neijts et al., 2014). Consistent with this, deletion of Cdx genes in the mouse embryo leads to truncation of the body axis; this can be rescued to some extent by exposure to Wnt or FGF signalling (Young et al., 2009; van de Ven et al., 2011; van Rooijen et al., 2012), further linking Cdx activity to the induction and/or maintenance of axial progenitors, which may include NMps.

### Insights from *in vitro* studies

**Fig. 5:**
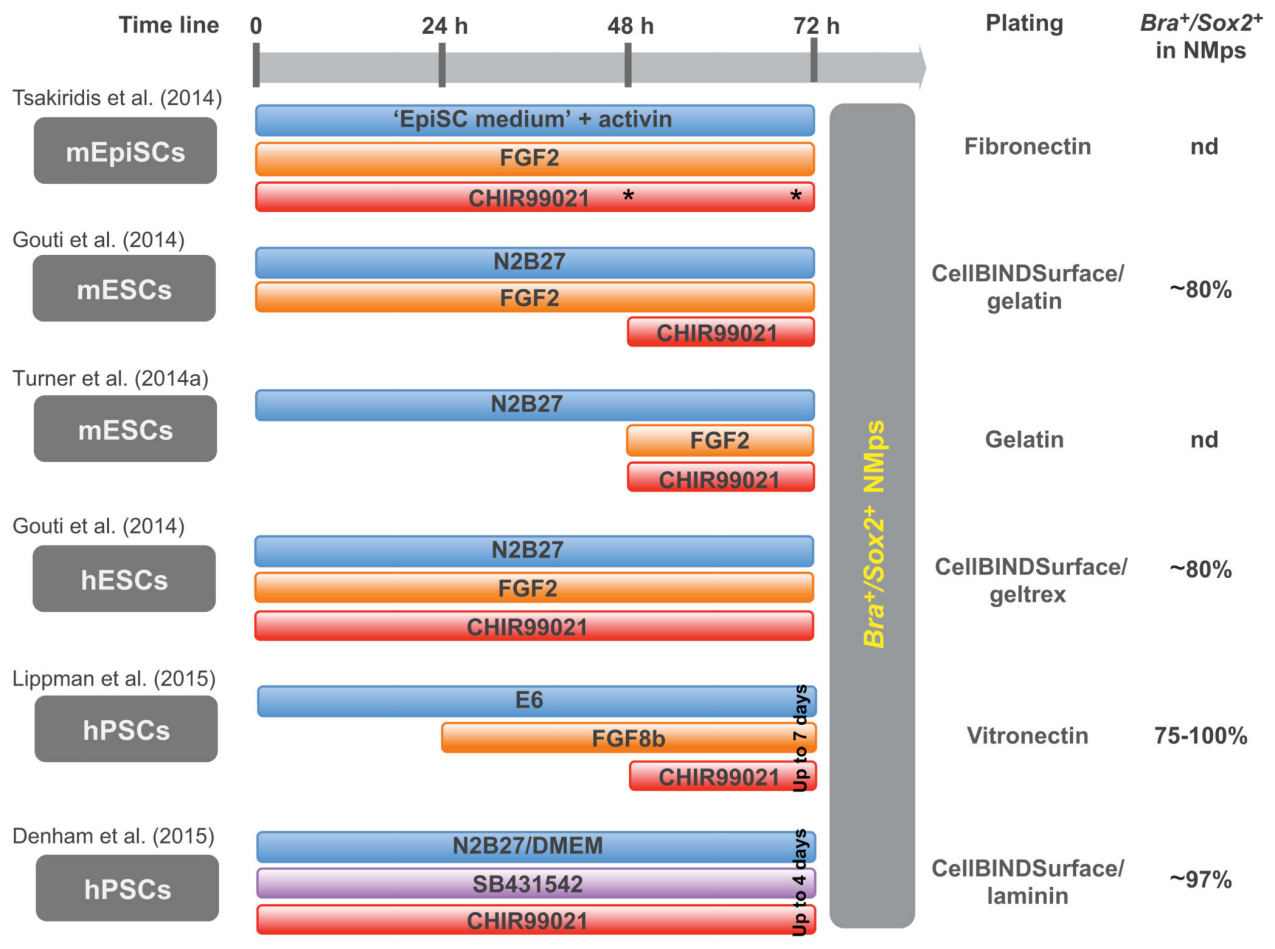
*In vitro* generation of NMps. Summary of protocols used in recent studies to generate NMps *in vitro* from pluripotent mouse or human cells. The application of exogenous molecules over time is detailed, as well as the matrix used to plate the cells. The percentage of Bra/Sox2 co-expressing cells observed in the NMp population is also indicated. Blue bars, medium base; orange bars, FGF regime; red bars, the addition of CHIR99021 (a GSK3β inhibitor, used for Wnt signalling activation); purple bar, the addition of SB431542 [an inhibitor of the activin receptor-like kinase receptors ALK4/5/7 (Acvr1b/Tgfβr1/Acvr1c)]. EpiSC medium refers to a DMEM-based medium containing activin A and FGF2. Note that Tsakiridis et al. (2014) obtained NMps after either 48 h or 72 h incubation in the differentiation regime (asterisks). Lippmann et al. (2015) maintained theNMp regime (FGF2+CHIR99021) for up to 168 h (7 days), generating progenitors with progressively more posterior identities. All studies varied/optimised culture conditions for the organism/cell line used. For detailed information about the individual protocols (including concentrations of exogenous molecules applied), refer to the original publications. m, mouse; h, human; ESC, embryonic stem cell; EpiSC, epiblast-derived stem cells; PSC, pluripotent stem cell; nd, not determined.

To better define the signals and molecular mechanisms regulating NMp formation, various laboratories have turned to more simple, *in vitro* cellular models, exploring the capacity of pluripotent cells to differentiate into multiple cell types. Recent reports from several labs have described the *in vitro* generation of cells that display functional characteristics of NMps. These experiments employ a common strategy (Fig. 5) that starts from cells exhibiting an epiblast-like state as a proxy for the embryonic epiblast from which NMps arise *in vivo*. In all cases, the activation of Wnt signalling at precise developmental time points (via the small molecule CHIRON99021, a GSK3β inhibitor) was crucial to generate NMps (Fig. 5).

An initial report (Tsakiridis et al., 2014) described the appearance of a population of Bra/Sox2-positive cells from mouse EpiSCs (maintained in the presence of activin and FGF2) following exposure to CHIRON99021 for 48 h (Fig. 5). This is a minor population that coexists with a larger population of mesendoderm progenitors (Bra^+^/Foxa2^+^), most likely induced by activin. Gene expression analysis confirmed activation of the Wnt pathway by CHIRON99021 and the upregulated expression of various lineage-affiliated genes, including endodermal, mesodermal and neural markers, together with a strong repression of the pluripotency genes *Oct4* (*Pou5f1*) and *Nanog*. In addition, known anterior neural markers such as *Pou3f2* were repressed, whereas posterior markers (*Zic3, Gbx2*) were induced.

Subsequent work demonstrated that the exposure of both mouse and human ESCs to FGF2 and CHIRON99021, in the absence of activin, led to more efficient generation of NMps, reaching up to 80% of the cells in culture (Gouti et al., 2014); a regime of two days of culture in the presence of FGF2 induced epiblast-like cells and a third day in the presence of FGF2 and CHIRON99021 generated NMps (Fig. 5). In a parallel study, Turner et al. (2014a) identified a responsive window (from day 2 to day 3 of mouse ESC differentiation) within which NMps can be induced by exposure to CHIRON99021; and this was more efficient when combined with FGF signalling (Fig. 5). These studies further demonstrated that NMps can subsequently be differentiated into neural fate by removing CHIRON99021 and FGF and replacing them with RA and a sonic hedgehog (Shh) agonist or into a mesodermal fate by maintaining CHIRON99021. This mesoderm differentiation regime recapitulates the effects described above of constitutively activating Wnt/β-catenin in *Bra*-expressing cells *in vivo*. However, as in the embryo, it is not simply the case that maintenance of Wnt signalling promotes mesodermal over neural fate in this context. For example, Gouti et al. (2014) demonstrated that *Bra* null ESC-derived NMps exposed to CHIRON99021 fail to make mesoderm, but can still form neural tissue. This is consistent with findings in the embryo that Wnt signalling is not incompatible with the generation of neural fates from NMps. The apparent multiple roles of Wnt signalling in caudal tissues require further investigation, and this new ability to generate NMps *in vitro* will now permit precise investigation of Wnt signalling in the control of NMp specification, maintenance and differentiation.

The experiments of Gouti et al. (2014) and Turner et al. (2014a) provide the first solid evidence for the dual-fated nature of *in vitro* generated NMps and, as noted above, this has been followed up by data which strongly suggest that single Bra/Sox2 co-expressing cells can generate clones containing neural and mesodermal cell types *in vitro* (Tsakiridis and Wilson, 2015). In addition, the Gouti et al. (2014) study characterised NMps and their derivatives through global gene expression profiling. We have compared their list of ~240 NMp-specific genes with other related data sets, including mouse genes expressed in the primitive streak in a Wnt3a-dependent manner (Dunty et al., 2014) and chick genes expressed specifically in the CLE/stem zone (Olivera-Martinez et al., 2014), as well as data from Tsakiridis et al. (2014) (supplementary material Fig. S1 and Table S1). These comparisons reveal interesting insights into the factors that direct NMp formation and differentiation (see supplementary material Fig. S1).

Importantly, Gouti et al. (2014) further showed that when epiblast-like cells are differentiated without exposure to Wnt (and so without an NMp intermediary step), this generated neural precursors with anterior rather than posterior identity, and our comparison of the transcriptional programmes underlying the generation of these two precursor populations at day 3 of the differentiation protocol reveals that they follow distinct developmental paths, with anterior precursors arising from a Wnt-less environment provided by the expression of multiple Wnt inhibitors (*Dkk2, Cer1, Sfrp1, Shisa3* and *Tcf3*). Each population also deployed different FGF ligand-receptor combinations, with NMps expressing *Fgf4*, *Fgf8* and the receptor *Fgfr1*, and anterior precursors expressing higher levels of *Fgf5*, *14* and *15*, and of *Fgfr2* and *3*. The two populations also appear to use distinct mediators of BMP inhibition; anterior neural precursors express higher levels of *Smad7*, whereas neural precursors derived from NMps have higher levels of *Smoc1*. A further distinguishing feature is the response to RA signalling, which promotes hindbrain and anterior spinal cord fates in anterior neural precursors, whereas neural precursors derived from NMps acquire more posterior spinal cord fates, expressing more 5′ Hox genes (Gouti et al., 2014).

In a more recent study (Lippmann et al., 2015), an almost pure population of BRA/SOX2-positive NMps was obtained from human ESCs, by allowing a day of rest following withdrawal of FGF2 and TGFβ1 and then exposing cells to an FGF ligand (FGF8b instead of FGF2) for 24 h, followed by culture with FGF8b and CHIRON99021 for up to 7 days (Fig. 5). The analysis of Hox gene expression at intervals during this latter period revealed that NMps sequentially activated more posterior combinations of Hox genes (see also Gouti et al., 2014), with expression of lumbosacral Hox genes (*HOXA/D10-12*) achieved by addition of the TGFβ ligand GDF11. Moreover, when NMps at different time points were exposed to RA, they downregulated *BRA* expression, entered neural differentiation and generated motoneurons with anterior-posterior identities according to the combination of Hox genes expressed at the time of RA addition. These findings thus support the model deduced from work in the embryo in which exposure to RA inhibits FGF/Wnt signalling and so arrests the temporal progression of 3′ to 5′ Hox gene expression, thereby setting the Hox code as differentiation commences (Diez del Corral and Storey, 2004).

Although these findings demonstrate that longer exposure to FGF and Wnt leads to the generation of more posterior neural tissue it is important to note that this can take place in response to the same regime even in the absence of *Bra* function, indicating that posterior identity can be conferred without mesoderm (Gouti et al., 2014). This is consistent with *in vitro* protocols that generate anterior neural tissue without an NMp intermediary, which can then be posteriorised to some extent by exposure to FGF/Wnt (Chambers et al., 2009; Peljto et al., 2010; Lupo et al., 2013; Meinhardt et al., 2014; Maury et al., 2015). However, the timing of exposure to such signals is critical for posteriorisation, as human ESCs induced to form anterior neural tissue by dual SMAD inhibition (Chambers et al., 2009) for 3 days did not exhibit posterior Hox gene expression in response to FGF/CHIRON99021 (Gouti et al., 2014). This suggests that posteriorisation must take place before or during neural induction (Gouti et al., 2014), and these events might be tightly linked in NMps, which serve to generate new neural progenitors throughout body axis elongation.

## When and where do NMps arise in the embryo?

As formulated above, one way in which NMps may arise in the embryo is from anterior neural plate that is subsequently exposed to the activity of posteriorising signals. In this scenario, NMps would have a shared lineage with neural cells that form the anterior CNS. The existence of clones that contribute to both anterior and posterior CNS, as well as to paraxial mesoderm, in the Tzouanacou et al. (2009) study is consistent with this hypothesis. However, these findings might simply reflect the labelling of cells in regions fated for both anterior and posterior CNS at very early epiblast stages and do not exclude the possibility of separate inductive events. Single-cell labelling in the early streak stage epiblast does indeed generate clones that contribute to both anterior and posterior CNS (Lawson and Pedersen, 1992). However, the analysis of clones from single epiblast cells directly labelled at later time points (Forlani et al., 2003) reveals that anterior and posterior lineages then become separate in the mouse embryo: epiblast cells at late streak to late streak/early bud (~E7.5) stages located rostral to the node generated neural-only clones that contributed to the more anterior hindbrain; by contrast, clones descended from epiblast cells closer to the node contributed to regions posterior to the hindbrain and included clones that contain both neural tissue and paraxial mesoderm. Furthermore, clones made in the anterior two-thirds of the epiblast at this stage map to the forebrain and midbrain (with few contributing to the hindbrain), but with no mesodermal contribution (Cajal et al., 2012). Together, these data indicate that lineages generating anterior and posterior CNS diverge at~E7.5 in the mouse embryo. As some of the cells that contributed to the spinal cord also contributed to paraxial mesoderm (Forlani et al., 2003), these data further indicate that NMps arise in an epiblast region that is spatially distinct from that which gives rise to anterior neural lineages (Fig. 1).

## To what extent do NMps contribute to the spinal cord?

It is important to determine the extent to which NMps contribute to the developing nervous system. Cell labelling studies in mouse embryos at headfold stages, when NMps are present in the embryo, have shown that some epiblast cells near the node can still give rise to neural-only clones in the hindbrain and anterior spinal cord. Many of these clones do not extend to the node (Forlani et al., 2003), suggesting that they are not part of a longer clone that might later include mesodermal tissue. Similar neural-only contributions are observed in the chick embryo following labelling of the CLE at headfold stages, where groups of one to three epiblast cells were shown to contribute to the hindbrain and anterior spinal cord and only few descendants encompass both neural and mesodermal lineages (Brown and Storey, 2000). These neural-only clones most likely reflect the continued contribution of anterior neural plate-derived cells, which must integrate and overlap with NMp-derived neural tissue in the anterior spinal cord. The precise position of this overlap could not be determined in the Forlani et al. (2003) study, as the clones were assessed after only ~24 h (i.e. neural-only clones might have continued more posteriorly if left for longer). However, it is also possible that neural-only clones reflect the activity of neural progenitors derived from NMps. Nonetheless, *Bra*-Cre-based lineage analysis indicates that the contribution of *Bra*-expressing cells to the neural tube begins in the anterior spinal cord, in the region approximately opposite somite 6 (Perantoni et al., 2005) (Fig. 1). This work further suggests that these cells initially contribute to ventral regions (see also Forlani et al., 2003; Cambray and Wilson, 2007; Anderson et al., 2013; Imuta et al., 2013) and that this comes to include more dorsal neural tube as axis elongation progresses (Perantoni et al., 2005; Chalamalasetty et al., 2014). Furthermore, Tzouanacou et al. (2009) found more neuromesodermal clones when they assessed embryos at later stages, indicating an increase in the NMp pool during the generation of posterior regions.

In summary, these findings in the mouse indicate that NMps generate ventral neural tissue at anterior spinal cord levels, where this is integrated with dorsal neural tissue derived from the anterior neural plate; however, the contribution of NMps to the neural tube becomes preponderant in the more posterior spinal cord, generating dorsal as well as ventral regions. Although detailed analysis of NMp contribution to spinal cord is currently lacking, it has been reported that ~65% of *Bra*-Cre-expressing cells are found in ‘trunk neural tube’ sections (Chalamalasetty et al., 2014). In addition, the majority of cells in the anterior primitive streak and adjacent epiblast co-express *Sox2* and *Bra* as the trunk is generated (Garriock et al., 2015) (see Fig. 3) and it is therefore likely that these cells are entirely responsible for the continued generation of new neural tissue as the body axis elongates.

## Are NMps induced independently of the anterior neural plate?

Although there are a number of mouse mutants that generate a ‘headless’ phenotype (e.g. Shawlot and Behringer, 1995), it has not been determined whether the trunk neural tissue that is generated transits through an initial anterior neural state or arises independently by a process involving the formation of NMps. One way to identify signals and mechanisms that underlie the formation of NMps is to investigate the ability to induce such cells in early epiblast cell populations. This has yet to be directly tested, but a number of experiments in chick embryos have addressed whether it is possible to generate posterior neural tissue without also inducing anterior nervous system. Up to the full primitive streak stage, grafts of the chick organiser/node juxtaposed with extra-embryonic epiblast are able to induce ectopic miniature neural tubes that express forebrain, midbrain, hindbrain and anterior spinal cord markers, but these studies did not assess posterior spinal cord markers (Waddington, 1932; Gallera, 1971; Dias and Schoenwolf, 1990; Storey et al., 1992). Older nodes (e.g. from the headfold stage) can induce hindbrain/ spinal cord without associated anterior neural markers in this assay (Storey et al., 1992). This could indicate that older nodes no longer produce anterior neural-inducing signals, but induce spinal cord directly. However, we cannot exclude the possibility that old nodes can induce neural tissue with an initial anterior character, which is then posteriorised. Whichever is the case, it will be important in future work to determine if signals from the node of any age can induce NMps and the posteriormost spinal cord.

The waning of neural-inducing signals in old nodes (Gallera, 1971; Dias and Schoenwolf, 1990; Storey et al., 1992) also suggests that any NMps in the transplanted node, or those induced by it, will quickly differentiate in the new ectopic context. This might reflect a necessity for other signals present in the embryo and/or a requirement for a critical mass of cells to generate/maintain a self-organising cell population capable of continued generation of new tissue (Turner et al., 2014a; van den Brink et al., 2014). Even if old nodes do not induce NMps, they can induce the expression of CLE/ PNT markers, such as *Nkx1.2* (Henrique et al., 1997). Explants of paraxial mesoderm from beneath the CLE can also elicit the expression of *Nkx1.2* in early neural plate explants without also inducing *Bra* expression (Delfino-Machin et al., 2005). These findings indicate that some aspects of establishing the CLE can be distinguished from induction of NMps.

If NMps are not readily induced by a grafted node, this might reflect differences between how this process normally takes place in the embryo and in this assay, in which grafts are juxtaposed with the extra-embryonic epiblast. It is possible, for example, that NMp specification is linked to mesoderm/primitive streak induction, and previous studies indicate that grafted nodes do not induce primitive streak (Dias and Schoenwolf, 1990; Storey et al., 1992; Beddington, 1994; Streit et al., 2000). Indeed, there is some evidence to link NMp formation with primitive streak induction; FGF-presenting beads induce *Bra* within 6 h in chick extra-embryonic epiblast and this is followed 4 h later by expression of the proneural gene *Cash4*, resulting in the appearance of a subset of cells that co-express Bra and *Cash4*, which arguably represent NMps (Storey et al., 1998). Thus, in the embryo, primitive streak induction, rather than anterior neural plate formation, might be a prerequisite for the specification of NMps. This would likely involve the creation of an appropriate signalling environment for NMps, with the provision of Wnt as well as FGF signals by the primitive streak.

## Relating NMp formation to epiblast patterning

**Fig. 6:**
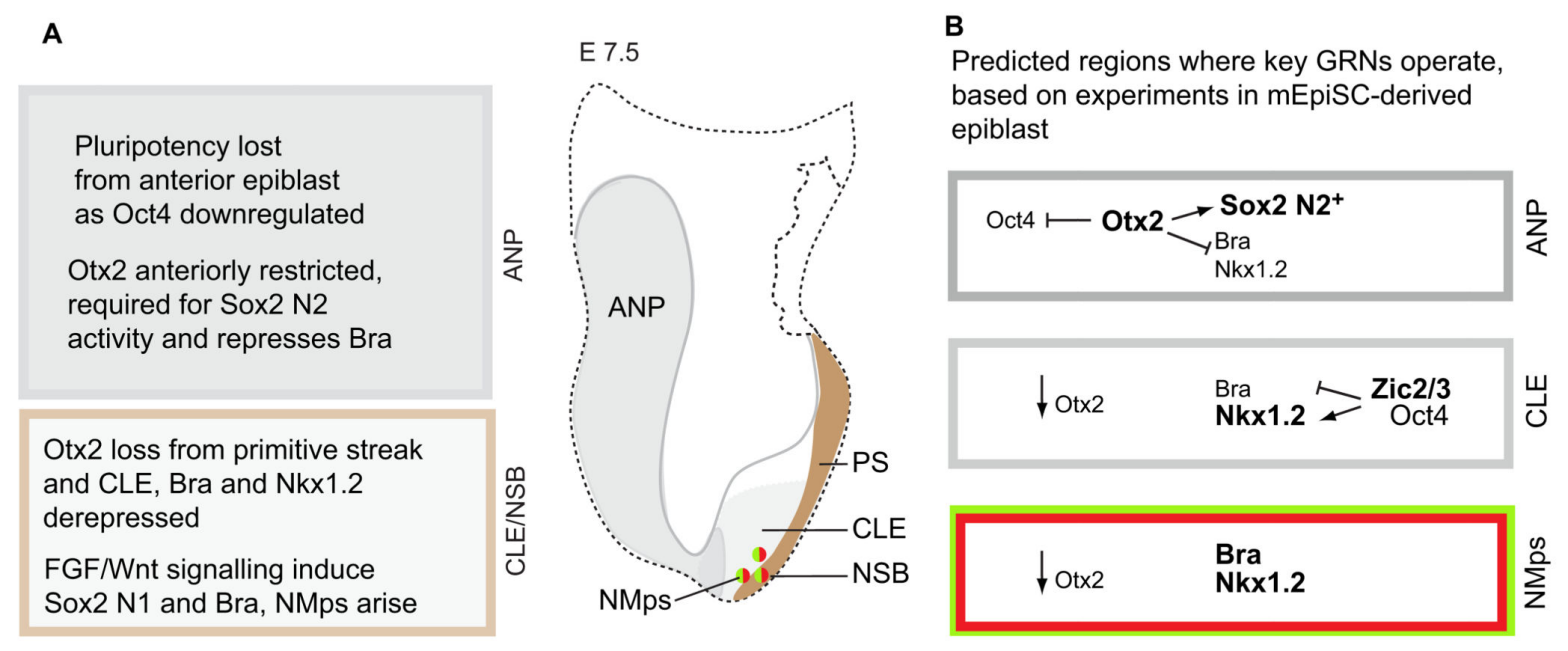
Summary of events contributing to the acquisition of neural fate in the anterior epiblast and to NMp formation. (A) Steps taking place in an E7.5 mouse embryo epiblast. The key steps leading to the acquisition of neural fate in the anterior neural plate (ANP; grey) and to NMp induction in the caudal lateral epiblast/node-streak border (CLE/NSB; light grey) are indicated. The primitive streak (PS) is also shown (brown). (B) The key gene regulatory networks (GRNs) predicted to be operating in each region, based on analyses in differentiating mouse EpiSCs (Iwafuchi-Doi et al., 2012).

It seems pertinent that NMps arise in the mouse embryo at about the time that the anterior epiblast finally loses pluripotency, which is determined by a decline in Oct4 levels (Osorno et al., 2012). This also coincides with restriction of the expression of the transcription factor *Otx2* to the anterior epiblast (Ang et al., 1994; Bally-Cuif et al., 1995); although *Otx2* is required in the underlying visceral endoderm for anterior neural plate induction, it is also needed in the epiblast to maintain anterior neural tissue (Rhinn et al., 1998; Kimura et al., 2000). More recent work further shows that, at E7.75 (the early headfold stage), Otx2 becomes responsible for *Sox2* N2 enhancer activity, specifically in the anterior neural plate (Iwafuchi-Doi et al., 2012). Together, these findings suggest that establishment of a neural state in the anterior epiblast takes place relatively late, as pluripotency is lost and as *Otx2* expression becomes anteriorly restricted, where it now acts to sustain *Sox2* N2 activity and specify forebrain and midbrain (Fig. 6A).

Using mouse EpiSC differentiation *in vitro* as a model system, Iwafuchi-Doi et al. (2012) have further defined the core gene regulatory interactions that occur during epiblast differentiation. Otx2 is also central to these actions: it works together with Sox2 to repress *Oct4* expression, and it can also inhibit expression of the CLE/PNT marker gene *Nkx1.2* (Iwafuchi-Doi et al., 2012). Extrapolated to the embryo, these data suggest that restriction of *Otx2* to the anterior epiblast establishes the anterior neural plate, but its downregulation in epiblast cells around the node may also derepress *Nkx1.2* and so concomitantly demarcate the CLE (Fig. 6B).

Importantly, *Otx2* is further found to repress *Bra* expression in differentiating mouse EpiSCs (Iwafuchi-Doi et al., 2012), and this might correspond to its action in the anterior primitive streak, where it is detected until late primitive streak stages. Indeed, *Bra* expression expands across the whole epiblast in *Otx2* mutant mouse embryos (Kimura et al., 2000). This potentially links *Otx2* downregulation in the primitive streak to NMp induction as well as establishment of the CLE. That *Otx2* downregulation is a prerequisite for NMp induction is further supported by the coincident onset of *Sox2* N1 enhancer activity in the primitive streak (Yoshida et al., 2014) (Fig. 6).

Iwafuchi-Doi et al. (2012) further found that the transcription factors Zic2/3 induce *Nkx1.2* but repress *Bra* in EpiSCs (Fig. 6). This condition is consistent with *Nkx1.2* expression, not just in NMps but also in neural progenitors in the CLE and PNT, potentially identifying further transcription factors that participate in the gene network regulating the transition of NMps to neural progenitors. In this process, the role of FGF-induced factors such as Churchill and Sip1, which inhibit *Bra* and promote neural fate in chick (Sheng et al., 2003), might also contribute to consolidate neural fate in cells that do not ingress through the primitive streak.

Together, these findings begin to build a molecular account of the regulatory steps in the early epiblast that underpin the establishment of the anterior neural plate and NMps (Fig. 6). The exact timings and molecular mechanisms underlying these interactions now need to be investigated and localised in distinct cell populations in the embryo. It will also be important to align these steps with the ‘preneural’ state identified in the chick embryo (reviewed by Foley et al., 2000; Streit et al., 2000; Stern, 2001) (Fig. 2) and with the transitions that occur during the emergence of mouse ESCs from pluripotency (Kalkan and Smith, 2014).

## Conclusions

Overall, the data reviewed here suggest a framework that extends Nieuwkoop’s activation-transformation model for the induction and patterning of the CNS (Fig. 2). This revised view involves induction of the anterior neural plate and its subsequent patterning to form posterior neural regions, including the forebrain through to the anterior spinal cord, but additionally incorporates the separate induction of an NMp population within the primitive streak-associated epiblast, which generates more posterior spinal cord. This proposal is based on evidence in chick and mouse embryos, which undergo extensive body axis elongation. NMps have yet to be reported in amphibian embryos and it might be that here the rapidly formed neural plate extends simply by convergent extension movements (Stern et al., 2006).

This NMp induction step appears separable from that of anterior neural plate induction, for the following reasons. (1) Anterior neural plate and NMp lineages diverge at late primitive streak stages prior to the establishment of neural fate in the epiblast. (2) The molecular mechanisms for making NMps are distinct from those that direct anterior neural plate; this is indicated by the different inputs that promote *Sox2* N1 (in NMps) and *Sox2* N2 (in anterior epiblast) enhancer activity. In the primitive streak, onset of N1 activity occurs as *Otx2* is downregulated, and is promoted by FGF and Wnt signalling, whereas in the anterior epiblast there is a switch to Otx2-dependent *Sox2* N2 activity. (3) NMp induction appears to be linked to primitive streak induction, as ectopic FGF can induce streak-like structures that include *Cash4*/Bra co-expressing cells. This conclusion is further supported by studies of the *in vitro* induction of NMps elicited by FGF and Wnt signalling, which would be provided by the primitive streak in the embryo.

Once established, NMps serve to extend the generation of new neural tissue until the end of body axis elongation, long after the node has lost its neural inducing ability, producing new neural progenitors that fuel the CLE. The production of neural and mesodermal tissue from this common precursor might then help to coordinate the differentiation and patterning of trunk tissues, as signals, such as RA, from the differentiating mesoderm then act back to repress FGF and Wnt signalling and promote the progression of neural differentiation (Diez del Corral et al., 2003; Wilson et al., 2009).

Altogether, these findings suggest that there are then two routes for making CNS neural progenitors: one involves the induction of the anterior neural plate and a second the induction of NMps in the primitive streak-associated epiblast, with a subsequent ongoing decision between neural and mesodermal fates. It will be interesting to determine what is shared and what is distinct about the molecular mechanisms that generate neural progenitors via these different routes. Further important questions are raised in this advancing area of research (see Box 2). In addition, the ability to create NMps *in vitro* will allow researchers to dissect more finely the molecular mechanisms that direct neural and mesodermal differentiation and will facilitate biochemical and genome-wide approaches, such as RNA-seq and ChIP-seq, that are currently challenging in embryonic cell populations. Finally, the *in vitro* generation ofNMps further opens up the possibility of investigating these processes using human pluripotent cells and exploring the potential therapeutic use of NMps (Box 1).

## Supplementary Material

Supplementary material available online at http://dev.biologists.org/lookup/suppl/doi:10.1242/dev.119768/-/DC1

Figure S1

Table S1

## Box 1 Potential applications of NMps

The *in vitro* derivation of NMps opens up a new experimental paradigm for studying the cellular and molecular basis of tissue generation. For example, *in vitro* derived NMps have already been used to define the scale and configuration of cell populations required for tissue self-organisation and generation (Baillie-Johnson et al., 2014; van den Brink et al., 2014). The use of NMps derived from human pluripotent cells in this context might also advance tissue engineering for therapeutic purposes. For example, NMps might prove particularly relevant for cell-based therapies as they passage poorly and differentiate quickly, and so present a low tumour formation risk. NMps may also be used to generate specific neuronal cell types with which to model spinal cord circuit development, such as lumbar motor neurons. Related to this, these *in vitro* approaches might facilitate the development of novel *in vitro* disease models, which can be used to analyse disease pathology and for small molecule screening. Finally, NMps derived *in vitro* from human cells will facilitate investigation of the fundamental biology of human spinal cord development.

## Box 2 Open questions

- Do NMps arise just once, early in development, or are they continuously produced in the epiblast region around the regressing node/anterior primitive streak?
- Are NMps a homogenous cell population or do different NMp types emerge at different developmental stages?
- Do some NMps self-renew throughout axis elongation or are all NMps transient?
- What is the relationship between pluripotency loss in the epiblast and the formation of NMps?
- To what extent do NMps make the posterior spinal cord – do they make all of it?
- What is common and what is distinct about neural progenitors generated via the anterior neural plate or through an NMp intermediary?
- To what extent do the gene regulatory networks operating in NMps identified *in vitro* operate *in vivo*?
