# Supplementary material for: Neuromesodermal progenitors and the making of the spinal cord
Source: Development. Author manuscript; Available in PMC 2016 Jul 24. (PMC4958456; doi:10.1242/dev.119768)
Supplement: Figure S1 [file NIHMS69120-supplement-Dev119768.pdf]

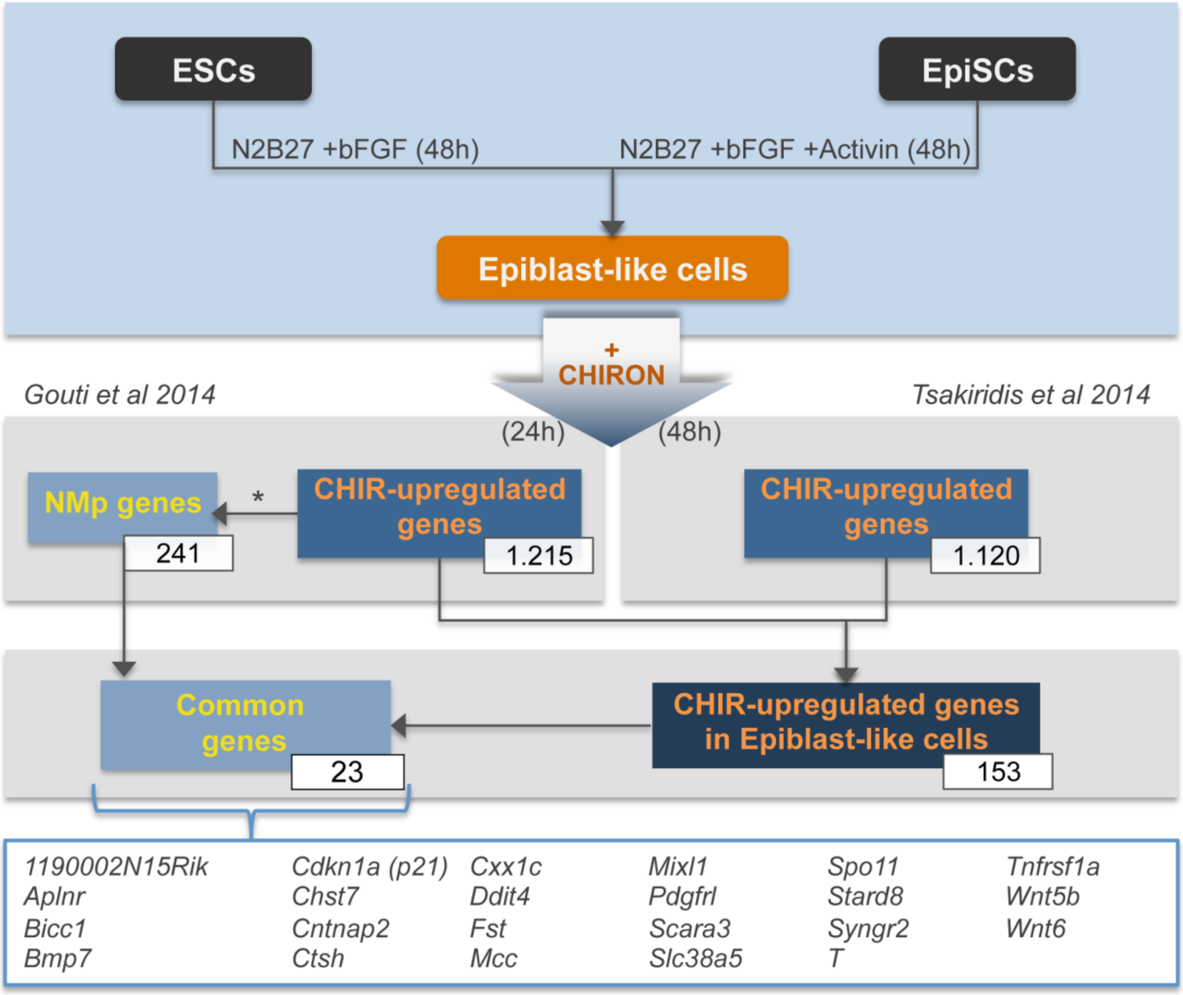


**Figure S1. Molecular insights into the gene expression profile of *in vitro* generated NMps.**

Comparison of the gene lists containing those upregulated by CHIRON99021 in mouse ESC-derived epiblast-like cells [24 h exposure, starting at day 2 of culture in N2b27+bFGF; 1.215 genes (Gouti et al., 2014)], and in mouse EpiSCs [48 h exposure in N2b27+bFGF+Activin; 1.120 genes (Tsakiridis et al., 2014)]. Gouti et al. (2014) identified 241 genes that were transiently upregulated by CHIRON99021 during the transition from epiblast to NMps, and then decreased when these cells embark on neural or mesodermal differentiation. Of these NMp genes, 26 are known to be expressed in the embryonic primitive streak in a Wnt3a-dependent manner (Dunty et al., 2014), and 16 have chick homologues that are specifically expressed in the CLE/stem zone and PNT (Olivera-Martinez et al., 2014) (see Table S1), supporting the specificity of the proposed NMp gene signature. A comparison (Table S1) of the two sets of genes upregulated by CHIRON99021 in either mouse ESC-derived epiblast-like cells or EpiSCs reveals a small overlap (153

genes). This likely reflects the higher cellular heterogeneity in the EpisSC-derived cultures, which were exposed to CHIRON99021 in the presence of Activin, and therefore generated not only NMps but also a larger population of FoxA2/T/Bra-expressing mesendodermal precursors [absent in the protocol employed by Gouti et al. (2014)]. Nonetheless, a key feature identified in both studies is the capacity of Wnt signalling to dismantle the pluripotency network, as shown by the strong downregulation of Nanog and Oct4 in CHIRON99021-treated EpiSCs.

**Table S1. Datasets comparison and annotations.**

Click here to Download Table S1
